# **β**-Cell Generation: Can Rodent Studies Be Translated to Humans?

**DOI:** 10.1155/2011/892453

**Published:** 2011-10-05

**Authors:** Françoise Carlotti, Arnaud Zaldumbide, Johanne H. Ellenbroek, H. Siebe Spijker, Rob C. Hoeben, Eelco J. de Koning

**Affiliations:** ^1^Department of Nephrology, Leiden University Medical Center, Postal Zone C3-P, P.O. Box 9600, 2300 RC Leiden, The Netherlands; ^2^Department of Molecular Cell Biology, Leiden University Medical Center, P.O. Box 9600, 2300 RC Leiden, The Netherlands; ^3^Hubrecht Institute, 3584 CX Utrecht, The Netherlands

## Abstract

**β**-cell replacement by allogeneic islet transplantation is a promising approach for patients with type 1 diabetes, but the shortage of organ donors requires new sources of **β** cells. Islet regeneration *in vivo* and generation of **β**-cells *ex vivo* followed by transplantation represent attractive therapeutic alternatives to restore the **β**-cell mass. In this paper, we discuss different postnatal cell types that have been envisaged as potential sources for future **β**-cell replacement therapy. The ultimate goal being translation to the clinic, a particular attention is given to the discrepancies between findings from studies performed in rodents (both *ex vivo* on primary cells and *in vivo* on animal models), when compared with clinical data and studies performed on human cells.

## 1. Introduction

Type 1 diabetes results from the specific destruction of *β*-cells by the immune system. The insulin-producing *β*-cells are the most abundant cell type residing in the islets of Langerhans, which are microorgans that are scattered throughout the exocrine tissue and represent only 1 to 2% of the total organ mass. *β*-cell replacement is considered the best therapeutic option, given the capacity of this particular cell type to accurately respond to highly variable changes in blood glucose level and as such to maintain glucose homeostasis. Islet transplantation via the portal vein of the liver is a promising approach and considerably less invasive than total organ transplantation. However, among other difficulties, the scarcity of donor material will always remain a major hurdle [1, 2].

Adult *β*-cell mass is known to be dynamic and to be able to respond to physiological changes in insulin demand such as obesity, pregnancy, and starvation. In theory, *β*-cell mass adaptation can occur via modification of cell size (hypertrophy versus atrophy) or modification of cell number (proliferation of existing mature *β*-cells or formation of new *β*-cells from progenitor cells versus apoptosis).

The origin of newly formed *β*-cells has long been debated [3–9], part of the observations reported remaining controversial and partial. In addition, many statements rely on studies performed in animal models or animal cell(s) (lines). As the ultimate goal is translation to the clinic, the aim of this paper will be to compare rodent and human data regarding postnatal cell types envisaged as potential sources to generate *β*-cells during the past decade. 

We will focus on the replication capacity of the *β*-cell itself then address the unexpected recent plasticity of differentiated cell types developmentally close to *β*-cells. For many years the dogma was upheld that terminally differentiated cells were committed to a specific function and could no longer change their identity. In contrast, progenitor/stem cells are expected to retain some multipotency capacities and therefore be more suitable for tissue replacement strategies. Nowadays more and more examples of efficient “transdifferentiation” have been reported, without an apparent need for a progenitor cell intermediate stage. Finally, given their various capacities, adult mesenchymal stem cells (MSC) are increasingly considered for clinical use in many different applications. We will review the recent reports about both pancreatic and extrapancreatic sources of MSC and the possible application of these cells for type 1 diabetes treatment.

Several groups reported on a successful derivation of both murine and human embryonic stem cells (ESs) to *β*-cell-like cells able to revert hyperglycemia in diabetic mouse models (for detailed review see [10]). Mimicking normal pancreatogenesis appears to be the best strategy to differentiate ES cells toward the endocrine lineage, although large differences in differentiation capacity exist between different ES cell lines using similar protocols [11]. Importantly, the risk of teratomas formation *in vivo*, inherent to ES cells when they remain undifferentiated, as well as ethical principles about any therapeutical use of human ES cells currently hold back extensive clinical applications of these cells. Recent developments on technology to generate induced pluripotent stem cells (iPS) hold great promise [12–16]. However, safety issues due to genetic and epigenetic abnormalities during reprogramming or in subsequent cell culture are a major hurdle for clinical transplantation. A more immediate application of ES and iPS cells is more likely to be their use in modeling human disease [17]. Therefore, in this paper, we chose to limit ourselves to alternative postnatal cell sources The data of this part are summarized in Table 1.

## 2. Generation of *β*-Cells from Mature Differentiated Cells?

### 2.1. *β*-cells

**Figure 1:**
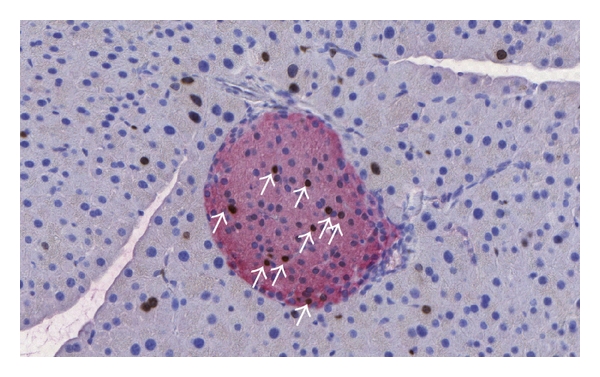
Section of murine pancreas stained with anti-insulin (red) and anti-BrdU (brown) antibodies. C57BL/6 mice were on a high-fat diet for 6 weeks. BrdU was injected for one week by s.c. injections, thereby labeling all proliferating cells. A number of replicating *β*-cells (arrows) and acinar cells are detected.

During adult life, *β*-cell replication appears to be a predominant mechanism of *β*-cell mass expansion in healthy mice (Figure 1). Dor et al. demonstrated that preexisting *β*-cells, rather than multipotent stem cells, are the major source of new *β*-cells during adult life and after partial pancreatectomy [18]. The study relies on a transgenic mouse model carrying a rat insulin gene promoter-controlled expression cassette encoding a tamoxifen-dependent CRE recombinase. A pulse treatment of tamoxifen irreversibly induced the expression of a reporter gene (human placental alkaline phosphatase (HPAP)) through CRE-mediated excision of a STOP sequence specifically in *β*-cells. Intriguingly, the percentage of HPAP-positive *β*-cells immediately after tamoxifen treatment and 4, 6, 9, and 12 months later remained very similar, indicating that during normal turnover in murine *β*-cells originate exclusively from preexisting *β*-cells. In islet injury models, either by pancreatectomy or by *β*-cell ablation using *β*-cell specific expression of Diphtheria toxin A resulting in 70% and 80% reduction in *β*-cell mass, respectively [18, 19], new *β*-cells appeared to be formed largely from genetically labeled preexisting *β*-cells and not from neogenesis or from expansion of non-*β*-cell precursors. Nevertheless, murine *β*-cell replication capacity appears to decline with age [20–22]. Furthermore, the question was addressed whether all *β*-cells contribute equally to growth and maintenance of *β*-cell mass or if distinct subpopulations exist. Label-retaining techniques were applied. Brennand et al. performed an *in vivo* pulse-chase labeling experiment using Histone 2B-GFP as reporter gene [23]. A uniform label across the entire *β*-cell population was observed. Next, a clonal analysis of dividing *β*-cells revealed that all clones were of similar sizes. Altogether this suggests that, in mice, the pool of *β*-cells is homogenous and all cells are able to replicate at the same rate. Therefore all *β*-cells can be candidate for *in vitro* expansion. Similar conclusions were obtained from a parallel study, in which a DNA analog-based lineage tracing method was developed to detect sequential cell division *in vivo* [24]. Recently Dor and colleagues further investigated the replication dynamics of adult murine *β*-cells and showed that replicated *β*-cells are able to reenter the cell division cycle shortly after mitosis. This short quiescence period of several days was found to be lengthened with advanced age [25] and shortened during injury-driven *β*-cell regeneration and following treatment with a pharmacological activator of glucokinase [25, 26]. The data of this part are summarized in Table 1.

In humans, Meier et al. investigated the *β*-cell mass from infancy to adulthood by performing immunohistochemistry and morphometric analyses on pancreas obtained at autopsy from 46 donors (from 2 weeks to 21 years of age) and determined pancreas volume by CT scan in 135 individuals of similar age [27]. The authors concluded that the predominant expansion of *β*-cell mass in humans occurs in early childhood (2.6% of *β*-cells positive for Ki67), without secondary growth phase of *β*-cell mass during adolescence, in contrast to rodents. Interestingly, the islets appear to increase in size rather than in number. Moreover, although the authors identified some *β*-cells (both individual cells and in small clusters) near the ducts, the number of these insulin-positive cells increased to a similar extent as the overall expansion of *β*-cell numbers in the islets, consistent with the hypothesis of *β*-cell replication. It is important to note that an efficient *β*-cell mass regeneration approach in a therapeutic perspective would require a similar rate of *β*-cell replication. Other studies confirmed these results. Kassem et al. reported that *β*-cell replication decreased progressively from 3.2% at 17–32 weeks of gestation to 1.1% perinatally [28]. After birth, levels of *β*-cell replication would drop further to reach less than 0.1% in young adults [29]. These data also correlate with the findings that *β*-cell mass is established by the first two or three decades of human life as determined by measuring accumulation of lipofuscin bodies as a marker to estimate *β*-cell longevity [30] or by *in vivo* thymidine analog incorporation and radiocarbon dating [31]. Remarkably, the adaptive increase of *β*-cell mass in adult humans appears to be modest in response to obesity (2- versus 10-fold) [32], as well as during pregnancy (1.4 fold versus 2 to 5-fold) compared to rodents [33, 34]. In contrast to rodents again, in both obese subjects and pregnant women, the adaptive increase in *β*-cell number was accompanied by an increased number of small new islets, indicative of neogenesis (cf. paragraph 2.3), rather than an increase in islet size or number of *β*-cells per islet, characteristic for *β*-cell replication. Finally a study performed on human pancreatic tissue collected from 13 patients who underwent a partial pancreatectomy showed that, unlike in rodents, a 50% pancreatectomy does not trigger any *β*-cell regeneration in adult humans. This corroborates with the high incidence of diabetes after partial pancreatectomy [35]. In summary, *in vivoβ*-cell replication capacity in humans appears to be mostly limited to the very early postnatal period, and the triggers of such a process are still unknown. 

Data available on *β*-cell proliferation *in vitro* are very limited when restricted to primary *β*-cells. An early report claimed that human *β*-cells were able to replicate efficiently (69% of BrdU/insulin double-positive cells) when exposed to a specific matrix (matrix produced by the rat bladder carcinoma cell line 804G) in the presence of hepatocyte growth factor/scatter factor (HGF/SF) [36]. However HGF induced a rapid decrease in insulin content [37]. This work was rapidly challenged by another report showing that the defined culture conditions were favorable for replication of ductal cells and not for *β*-cells [38]. In a comparative study between *in vitro* proliferation of purified human and rat *β*-cells, Parnaud et al. also failed to detect any *β*-cells that were positive for Ki67 or had incorporated BrdU even after 10 days of exposure [39]. However a clear proliferation of purified rat *β*-cells was observed and could be further enhanced by defined coatings or growth factors. Proliferation of human *β*-cells in (intact) isolated human islets was also assessed in the same study and remained undetectable. Therefore it appears that culture conditions for efficient human *β*-cell replication *ex vivo* have not been clearly identified.

### 2.2. *α* Cells

Differentiation of endocrine non-*β*-cells to *β*-cells is an interesting alternative mechanism for increasing the *β*-cell mass. A limited number of studies supported this new concept. Collombat et al. showed in various transgenic mouse models that forced expression of Pax4 at different stages (in pancreatic progenitor cells, endocrine precursor cells, or in mature *α* cells) resulted in a shift of all endocrine lineages toward a *β*-cell fate [40]. A bicistronic vector (Pax4-IRES-*β*-galactosidase) was used in order to follow cells of interest by staining for *β*-galactosidase activity. Importantly, the authors observed an age-dependent increase in islet size and in number of insulin/*β*-galactosidase double-positive cells and a concomitant decrease in *α*-cell content. These observations indicate that ectopic Pax4 expression can also force conversion of adult glucagon-expressing cells into *β*-cells. Remarkably, the subsequent decrease in glucagon was found to activate the differentiation of ducts-associated progenitor cells *α* cells (via an intermediate stage of Ngn3 positive cells). However the newly formed *α* cells failed to correct the hypoglucagonemia since they were shown to be rapidly converted into *β*-cells upon Pax4 ectopic expression. Notably, the expression of Pax4 in glucagon-positive cells has been reported to be sufficient to restore a functional *β*-cell mass in diabetic mice, although only in the young animals. Thorel et al. developed a transgenic mouse model of near total *β*-cell ablation that relies on the specific expression of Diphtheria Toxin Receptor (DTR) in pancreatic *β*-cells (expression driven by the rat insulin promoter) [41]. After administration of Diphtheria Toxin, a rapid and extreme (>99%) *β*-cell destruction by apoptosis was observed resulting in a characteristic diabetes within a couple of weeks. If given insulin, the mice survived and showed a slow *β*-cell mass regeneration. Only after 5 months the regenerated *β*-cell mass was able to maintain glucose homeostasis without exogenous insulin administration. Using a doxycycline inducible *α*-cell lineage tracing system, the authors established that one month after *β*-cell ablation, a large but variable (32 to 81%) fraction of newly formed *β*-cells resulted from the transdifferentiation of about 5 to 10% of *α* cells. Importantly, almost all (~90%) were bihormonal (still expressing glucagon even as far as 10 months after ablation). Of note, no *β*-cells were found in extrainsular locations. Therefore, in contrast to previous studies [19], the *β*-cell mass regeneration was attributed to neogenesis through *α*-cell transdifferentiation, rather than to the slow self-replication mechanism of preexisting mature *β*-cells. The authors suggested that the amount of *β*-cell loss and the type of injury would determine the mechanism of regeneration. This theory of unexpected pancreatic cell plasticity triggered by extreme *β*-cell loss was supported by a parallel study done by Chung and colleagues [42]. The model used was a combination of pancreatic duct ligation (PDL) with specific elimination of preexisting *β*-cells by alloxan [42]. No lineage tracing method was used in this work, and results are based on immunohistochemistry stainings performed at 7 and 14 days. The authors reported a rapid regeneration of *β*-cell mass within weeks and suggested that the newly formed *β*-cells resulted mainly from conversion of adult *α* cells. Interestingly the authors also observed that under injury conditions, *α* cells were able to replicate. However no *α*-cell division was required for conversion into *β*-cells. Importantly the regenerated *β*-cell mass was not sufficient to revert hyperglycemia, possibly due to the persistent inflammation caused by the PDL. In summary, three independent studies performed in transgenic mouse models showed that *α*- to *β*-cell conversion can occur. One could wonder how this relates with the developmental process. In rodents, the glucagon gene is expressed in the earliest endocrine cells that can be detected [43]. However, Herrera et al. demonstrated that, during murine embryogenesis, mature glucagon- and insulin-producing cells share a common precursor but belong to separate developmental lineages [44]. 

In human cells, the embryologic situation appears to be different, since insulin-positive cells are the first to be detected during development [45]. Therefore it is not known whether this extraordinary islet cell plasticity exists in adult human *α* cells *in vitro* or *in vivo*. If so, *α* cells, especially if their capacity to replicate under injury condition is confirmed, could be an ideal intraislet source for *in vivo* regeneration of *β*-cells.

### 2.3. Ductal Cells

Pancreatic ductal cells have long been thought to be the main source for progenitor cells in the pancreas (cf. reviews [3, 8]). 

Several rodent studies based on immunohistochemical observations in animal models, suggested a possible mechanism of islet neogenesis via recapitulation of the embryological development after activation and differentiation of ductal progenitors [46–50]. Recently a number of lineage tracing studies in transgenic mouse models have been performed in order to identify putative endocrine progenitors in adults, but provided contradictory conclusions. Bonner-Weir and collaborators made use of the human carbonic anhydrase II (CAII) promoter, specific for differentiated ductal cells, combined to a tamoxifen inducible CRE-ER/Lox recombination system [51]. CAII-expressing cells within the pancreas acting as precursor cells gave rise to both pancreatic endocrine and exocrine tissues after birth and after injury. After PDL 25% of *β*-cells were labeled. The authors proposed that ductal cells represent a pool of homogenous cells from which a fraction can dedifferentiate into progenitor cells that are able to regenerate both endocrine and exocrine tissues. In a parallel study, Heimberg and colleagues suggested a slightly different model: a rare subpopulation of endocrine progenitor cells, located in the ductal lining, could be activated upon PDL in adult mouse pancreas and subsequently started to express an embryonic key endocrine developmental factor, Ngn3 [52]. Differentiation of an Ngn3-positive subpopulation gave rise to all islet cell types, including glucose responsive *β*-cells both in situ and when cultured in embryonic pancreatic explants. However, in a subsequent study a lineage tracing of HNF1*β*-positive ductal cells was performed. 65% of pancreatic ductal cells were labeled. The authors show that the ductal epithelium does not make a significant contribution to acinar or endocrine cells during neonatal growth (6-month observation period) or upon regeneration condition (PDL or Alloxan followed by EGF/gastrin treatment) [53]. Of interest, earlier lineage studies by Melton's group pointed out on the heterogeneity in developmental potential among “duct-like structures” in early embryo [54]. Lineage tracing experiments suggested that Ngn3-positive cells are indeed islet progenitors but distinct from duct progenitors. On the other hand, the authors could not rule out the possibility that a minor population of mature ductal cells is able to transiently activate Ngn3 gene expression and subsequently contributes to islets neogenesis. Lineage tracing of Muc1 ductal/acinar cells confirmed that Muc1-positive cells give rise to endocrine cells in utero, in line with embryological development [55]. However, after birth, Muc1 lineage-labeled cells were found to be restricted to the exocrine compartment, with no detectable contribution to islet cells. No injury or regeneration models have been tested in that particular model. Along the same line, another independent lineage labeling using the Sox9 promoter (active in early pancreatic progenitors) and resulting in 70% of labeled pancreatic ductal cells established that very few nonendocrine cells continue to arise from Sox9-positive precursors in early postnatal life, but no endocrine or acinar cell neogenesis from Sox9-positive cells occurs during adulthood [56]. Intriguingly, in contrast with an earlier study [52], the authors found that after PDL, Sox9-positive cells give rise to Ngn3-positive cells but these cells do not contribute to islets. Thus, although the function of ductal cells as endocrine progenitors in embryonic development is commonly accepted, more insight is needed into the potential of cells from the ductal compartment as an alternative source for the formation of new *β*-cells in adult rodents.

**Figure 2:**
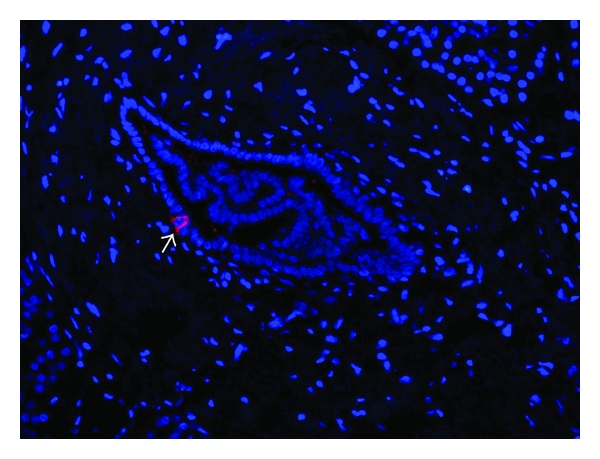
Section of human pancreatic exocrine tissue stained with an anti-insulin antibody (red). Nuclei are stained by DAPI (blue). An insulin-positive cell (arrow) is detected in the duct lining suggesting a process of *β*-cell neogenesis.

Immunohistochemistry stainings performed on tissues obtained at autopsy or after pancreatectomy are obviously the only possible technique to evaluate the *β*-cell mass in humans. The occurrence of neogenesis is suggested by the presence of insulin-positive cells in the ductal area combined to the absence of replication of this cell population (ruling out the expansion of a preexisting insulin-positive ductal cell population) (Figure 2). Careful quantification and repeated observations in different contexts can result in strong indications. Meier et al. analyzed pancreatic tissues obtained at autopsy from 46 children aged from 2 weeks to 21 years and observed about 0.5% of insulin-positive ductal cells [27]. A similar percentage has been reported by Reers et al. that studied pancreatic tissues from 20 donors aged from 7 to 66 years [57]. The rate of islet neogenesis does not seem to be affected by aging. Importantly, Butler et al. observed that ductal cells positive for insulin were increased by 2- and 3-fold during obesity and pregnancy, respectively [32, 33]. Therefore it appears that in humans islet neogenesis from ductal cells is stimulated during metabolic situations that require an increase in insulin level. It should be noted that during both obesity and pregnancy the adaptive increase of *β*-cell mass is much more limited in humans compared to rodents. In pathophysiological condition such as chronic pancreatitis, Phillips et al. also observed a significant increase of insulin-positive ducts in adult human pancreas of 11 patients compared to control group [58]. In another study, the question was addressed whether *β*-cell neogenesis occurs in the transplanted pancreas of type 1 diabetic patients who had received a simultaneous pancreas-kidney transplant (SPK) [59]. Pancreatic tissues from 9 SPK patients and 16 nondiabetic organ donors were examined by immunohistochemistry. Remarkably, high numbers 33 to 90% of ductal cells were found to be insulin positive, and 17% to 95% of the ducts harboured insulin-positive cells in SPK patients with recurrent autoimmunity and diabetes. A high degree of neogenesis was observed in patients with the most severe *β*-cell loss, whereas a low degree of neogenesis was observed in normoglycaemic patients, suggesting that in the particular context pancreas transplantation, hyperglycemia and chronic inflammation may strongly stimulate *β*-cell neogenesis from ductal cells. Intriguingly, a study by Bouwens et al. analyzing pancreatic tissues from nine adult donors revealed that 15% of all *β*-cells were located in units with a diameter less than 20 mm and without associated glucagon-, somatostatin-, or pancreatic polypeptide cells [29]. These single *β*-cell units were situated in or along ductules, from which they appear to bud as previously noticed in fetal and neonatal pancreas. Furthermore, simultaneous presence of Ki67-positive ductal cells (0.05%) and absence of Ki67-immunoreactive budding *β*-cells suggested that *β*-cell neogenesis depends on ductal cell proliferation and differentiation in humans. 

About ten years ago murine ductal cells cultured *in vitro* were reported to form 3D clusters that differentiate to functional islet cells, which are able to respond to a glucose challenge and to reverse diabetes in mice [60]. An interesting strategy for the prospective isolation of putative progenitors from an enriched ductal cell population is also being pursued by Taniguchi and colleagues [61, 62]. The approach combines immunohistochemical analysis of mouse pancreas to define new phenotypic markers and flow cytometry cell sorting to isolate clonal cell populations that are able to differentiate toward the endocrine lineage *in vitro* or *in vivo*. The data suggest that a population of progenitor cells was present among CD133-positive ductal cells. 

Koblas et al. confirmed that a subpopulation of CD133-positive cells in human islet-depleted tissue was able to differentiate into functional insulin-producing cells *in vitro* and to secrete insulin in a glucose-dependent manner [63]. Furthermore, Bonner-Weir and collaborators showed that human primary ductal cells could be isolated from islet-depleted pancreatic tissue, expanded in culture, and triggered to differentiate towards glucose responsive islet-like clusters [64]. These results were confirmed by Gao et al. who further characterized the nature of these pancreatic progenitor cells [65]. During monolayer expansion, two subpopulations of proliferating cells were observed, CK19-positive ductal cells at an early time point (day 3) and nestin-positive cells at a later time point (day 7). Under serum-free conditions and Matrigel covering of the cells, the CK19-positive cells, but not the Nestin-positive cells, were able to form islet-like clusters that contain insulin- and glucagon-positive cells. When transplanted under the kidney capsule of nude mice, one out of five grafts demonstrated further growth with foci of both endocrine and exocrine cells. Next, Bonner-Weir and colleagues used magnetic cell sorting and antibodies raised against the ductal surface marker CA19-9 to isolate ductal cells from islet-depleted tissue [66]. Transplantation experiments of purified ductal cells versus unpurified preparations (56% CK19-positive cells only) into normoglycemic NOD/SCID mice revealed that differentiation of ductal cells to insulin-producing cells was dependent on the presence of nonductal cells, probably pancreatic stromal cells as suggested by the authors. Of interest, *in vitro* islet-to-duct plasticity has also been reported for human cells [67, 68].

Although some lineage tracing studies in rodents have provided contradictory results, most *in vivo* and *in vitro* data from both human studies indicate that cells from the ductal compartment are an attractive putative cell source for *β*-cell replacement strategies.

### 2.4. Acinar Cells

Besides the ductal origin hypothesis, a number of studies show that acinar cells might also display a certain degree of plasticity. 

Melton and colleagues suggested that acinar cells and endocrine cells share a common progenitor after the ductal cell lineage has already separated [54]. On the other hand, Bouwens and colleagues proposed a variant of the ductal origin hypothesis, in which acinar cells would be able to transdifferentiate towards *β*-cells, through an intermediate dedifferentiated ductal-like stage [49, 69]. This model was supported by more reports. *β*-cell mass regeneration has been studied after streptozotocin treatment in a transgenic mouse model expressing interferon gamma under the control of the insulin promoter [70]. New *β*-cells appeared to result primarily from the formation of new islets from small pancreatic ducts. However, interestingly, some putative transitional cells were identified harboring both exocrine and endocrine granules, indicative of acinar cells as possible precursor cells. Similar observations were made in a parallel study after PDL [71]. Furthermore, in order to visualize better possible intermediate stages during acinar-to-ductal transdifferentiation in a PDL injury model, Lardon et al. took advantage of the fact that dexamethasone treatment inhibits the loss of amylase from acinar cells [72]. Putative transitional cells coexpressing acinus-specific (amylase) and duct-specific (CK20) markers were identified *in vivo*. Furthermore, acinus-to-islet conversion was confirmed *in vitro* after isolation of acini and identification of putative transitional cells coexpressing acinus-specific (amylase) and *β*-cell-specific (insulin) markers. Several groups recently performed some *in vivo* lineage tracing analyses using acinus-specific promoters (amylase and elastase). Replication of preexisting acinar cells is seen as the major mechanism for regeneration of the acinar tissue. Moreover, acinus-to-duct transdifferentiation has been reported to occur *in vivo*, although at a very low frequency, in mouse models for pancreatitis [73], and in a mouse model that develops insulin-positive cell-containing hyperplastic ducts in response to the growth factor TGF*α* [74]. However, the same authors also showed that the insulin positive cells adjacent to acinus-derived ductal cells arose from preexisting insulin-positive cells and not from acinar cells. Along the same line, Stoffers and collaborator failed to observe any acinus-to-*β*-cell transdifferentiation in adult mice under normal conditions and after 70% pancreatectomy, PDL, or caerulein-induced pancreatitis [75]. On the other hand, Melton et al. reported that adenovirus-mediated coexpression of Pdx1, Ngn3, and MafA *in vivo* in pancreas from adult mice was sufficient to induce the transdifferentiation of mature exocrine cells into *β*-cells that are indistinguishable from endogenous islet *β*-cells in size, shape, and ultrastructure [76]. However the question remains open whether acinar cells or other possible precursor cells were reprogrammed in these experiments.

*In vitro*, rat exocrine cells treated with dexamethasone can convert to hepatocyte-like cells. In contrast, when cultured in low serum medium (1%) in the presence of EGF and LIF, rat exocrine cells can transdifferentiate to functional *β*-cells [77]. In that particular study, 10% of dedifferentiated acinar cells expressed insulin, with a total insulin content of 40 to 90% of primary *β*-cells, and transplantation of about 100,000 of these insulin-positive cells was sufficient to revert hyperglycemia in a diabetic nude mouse model. In an independent study, Minami et al. showed that suspension culture in presence of EGF and nicotinamide converted 5% of adult murine acinar cells to glucose responsive insulin-producing cells [78]. An adenovirus mediated *in vitro* lineage tracing study using the acinus-specific amylase or elastase promoter confirmed the identity of the starting population. In addition, acinus-to-duct transdifferentiation was shown to occur, in response to EGF-receptor signalling, through an intermediate nestin-positive stage in an *in vitro* culture of pancreatic explants [79].

Regarding human cells, there are no data available about a possible acinus-to-islet cell plasticity. One possible explanation is the selective death by apoptosis of human acinar cells when cultured *in vitro*. In contrast, human ductal cells can survive, adhere to plastic culture dish, and proliferate [80]. Nevertheless the possible acinus-to-duct transdifferentiation suggested by others [81] cannot be ruled out in this study.

### 2.5. Liver Cells

Liver and pancreas have a common embryological origin, both arising from the primitive gut. Therefore liver cells have been hypothesized to constitute a potential cell source for *β*-cell generation [82]. 

Ferber and collaborators demonstrated that an adenovirus-mediated expression of the key pancreatic transcription factor Pdx1 in mouse liver (intravenous infusion) resulted in transdifferentiation of hepatocytes to *β*-cell-like cells [83]. Pdx1 expression found in 60% of hepatocytes resulted in a 3-fold increase of plasma insulin levels, 59% as insulin and 41% as proinsulin. These data indicate that proinsulin was processed which was substantiated by expression of the prohormone convertase 1/3. The amount of insulin produced was sufficient to reduce hyperglycemia in a diabetic mouse model. A similar approach was successfully reported with adenovirus-mediated expression of Pdx1 or Ngn3 [84] and the coexpression of NeuroD1/Beta2 and betacellulin [85] in the liver. Of note, although the overexpression of Pdx1 was able to revert the elevated blood glucose of diabetic mice, the animals died from liver inflammation most likely due to the exocrine-differentiating activity of Pdx1. Interestingly Yechoor et al. showed that gene transfer of a pancreatic key transcription factor (Ngn3 in this study) in liver leads to long-term diabetes reversal in mice [86]. However the authors demonstrated that, although insulin expression was transiently induced in terminally differentiated hepatocytes, the long-term diabetes reversal obtained in these mice was resulting from the differentiation of hepatic progenitors able to generate islet-like clusters. The phenomenon was described as “transdetermination,” that is, lineage switching in lineage-determined, but not terminally differentiated, cells.

Human adult liver cells were shown to expand *in vitro* and to transdifferentiate towards an endocrine pancreatic lineage after Pdx1 overexpression [87]. Pdx1-expressing human liver cells were found to express insulin that is stored in secretory granules, which are released in a glucose-regulated manner. When transplanted under the kidney capsule of diabetic immunodeficient mice, these cells ameliorated hyperglycemia for prolonged periods of time. Similar studies using human fetal progenitor liver cells were reported [88, 89]. Since harvesting and propagating significant numbers of primary hepatocytes from patients with diabetes would be theoretically feasible, the liver can be considered as an interesting extrapancreatic source for *β*-cell replacement therapy.

## 3. Generation of *β*-Cells from Adult Mesenchymal Stem Cells?

Mesenchymal stem cells (MSC) were originally identified in bone marrow by Friedenstein in 1976 as a rare, heterogeneous, non hematopoietic, and multipotent stromal population able to differentiate to mesenchymal lineages including bone, fat, and cartilage. MSC are virtually present in all organs, including the pancreas and the islets of Langerhans. It is now generally accepted that the perivascular area harbors the MSC, explaining the ubiquitous distribution of these cells in the body [90]. Interestingly, MSC can be obtained from live donors (and potentially from the patient itself) and are easily expandable *in vitro*. Therefore, despite the fact that their identity and their exact role *in vivo* have not been clearly defined yet, mesenchymal stem cells have been envisaged for a broad range of therapeutic applications including type 1 diabetes [91, 92]. The data of this part are summarized in Table 2.

### 3.1. Islet(-Derived) MSC

We and others reported on the expansion of MSC-like cell population from isolated human islets: human islet-derived precursor cells (IPC) [93–95], NIP/Nestin-positive Islet-derived Progenitors [96], PIDM/Pancreatic Islet-Derived Mesenchymal cells [97], PHID/Proliferating Human Islet-Derived cells [98], and more [99, 100]. These cells are able to proliferate *ex vivo* and can be passaged. Interestingly, most of the groups reported a common characteristic, which is aggregation into clusters ranging in sizes between 50 and 200 *μ*m (similar range as primary islets) under serum starvation and a subsequent increase in expression of endocrine markers. However others failed to reproduce these data of partial differentiation [101, 102]. In any case, the endocrine markers remained at very low level when compared to freshly isolated human islets. Davani et al. reported a further capacity of IPC to differentiate into functional *β*-cells that secrete human C-peptide in response to glucose after reimplantation of 4-day-old aggregates in mice [94]. 

The origin of these cells remains elusive and very controversial. Initially Gershengorn and colleagues proposed an Epithelial-to-Mesenchymal Transition (EMT) to occur from human *β*-cells [93]. However this idea was rapidly refuted by the same group and others after lineage tracing experiments performed in transgenic mouse models that failed to show any EMT of murine *β*-cells [103–106]. The overall conclusion of these four studies was that the proliferative cell population derived from cultured murine islets was not originating from *β*-cells, since no *β*-cell specific markers were identified in these cells. Efrat and collaborators recently developed a lineage tracing system similar to the techniques applied in transgenic mouse models, but now applied to human cells *in vitro* using lentiviral vectors [107]. The dual viral system relies on the *β*-cell specific expression of the CRE recombinase in one vector and a CMV-GFP reporter vector in another vector in which GFP expression is restricted by a “floxed” intermediate sequence. As lentiviral vectors integrate into the genome of the transduced cells, the reporter gene will remain expressed in all cells originating from the initial pool of labeled cells. In their follow-up study, Russ et al. slightly modified the system by using a tamoxifen-inducible CRE/ER recombinase, restricting the labeling period to the duration of a short (overnight) tamoxifen pulse [108]. Human *β*-cells were efficiently (50%) and specifically labeled by the dual lentiviral system. This powerful technique provided evidence that human *β*-cells can dedifferentiate to an MSC-like cell population and proliferate when cultured *ex vivo*, in contrast to mouse *β*-cells, as revealed earlier from the transgenic mice studies [103–106]. Intriguingly, 40% of cells exhibiting MSC markers in culture (likely to be the cell population previously named IPC, NIP, PIDM, or PHID by others) resulted from an EMT of *β*-cells. On the other hand, several groups suggested, but never unequivocally demonstrated, the presence of a mesenchymal stem cell population within human islets [94, 96, 97]. In a recent study, we investigated the presence of MSC(-like cells) in freshly isolated human islets, and we identified a double-positive CD90/CD105 population representing approximately 2% of the total islet cell population. The presence of these cells inside freshly isolated human islets was confirmed by confocal microscopy [95]. An independent study validated the presence of pancreatic MSC in the periacinar, perivascular, and periductal space of human pancreas [109]. The functional significance of the presence of these cells in the islets and the possible interplay between islet-MSC, endocrine cells, and the vascular system in human islets remain to be further clarified. Altogether these data suggest that the MSC-like cell population derived from human islets in culture results from subpopulations of at least two origins: proliferation of islet-MSC and proliferation of dedifferentiated *β*-cells. More studies, in particular detailed lineage-tracing experiments will be needed to confirm this model. 

At the moment it remains unclear whether all mesenchymal stem(-like) cells are equal and whether MSC originating from islets would be more prone to differentiate toward the endocrine lineage. Two groups determined the gene expression profiles in human islet-derived MSC [110, 111]. Both studies confirmed the common mesenchymal character of the population with the archetypical bone marrow-MSC. Remarkably cultured islet-derived IPCs are different from bone marrow-MSC (BM-MSC) in that they express a set of islet-specific genes, although at low level. Upon differentiation, following the rather basic differentiation protocols developed so far for these cells, gene expression data showed that IPCs are able to go further along the endocrine pathways than BM-MSC.

In summary, islet-(derived) MSC could be of a valuable therapeutic significance since they appear to retain some genetic characteristic making them closer to endocrine cells than other sources of MSC such as bone-marrow-derived MSC. Further studies will be needed to verify this hypothesis. Furthermore, the observation that human *β*-cells can dedifferentiate *ex vivo* into a mesenchymal phenotype in contrast to murine *β*-cells illustrates again the discrepancy in plasticity of these cells between rodents and humans.

### 3.2. Exocrine/Islet Depleted Tissue(-Derived) MSC

Similarly to human islet cells cultured *ex vivo*, two independent studies reported that a population of MSC(-like) cells could be derived from human pancreatic exocrine tissue. Under defined conditions, these cells could show some sign of differentiation toward the endocrine lineage [112, 113]. 

The exact origin of these cells remains also unclear. On the one hand, epithelial-to-mesenchymal transition from exocrine cells has been proposed by Seeberger et al. [114]. Along the same line, Fanjul et al. described coexpression of ductal markers and mesenchymal markers both in cultured human ductal-like cells *in vitro* and in ductal cells in one pancreas from a nondiabetic subject and three pancreata from patients with type 2 diabetes [115]. Shin et al. demonstrated that the transdifferentiation capacity of human ductal cells was reduced after EMT [116]. On the other hand, Sordi et al. suggested that the mesenchymal stem cell population growing out of cultured pancreatic tissue (both endocrine and exocrine fractions were tested) would be mostly originating from proliferating “pancreatic MSC” that are partly derived from the bone marrow [109]. Total bone marrow transplantations from donor GFP transgenic mice in lethally irradiated recipient mice were performed. Surprisingly, after 12 weeks, GFP-labeled bone-marrow-derived cells were found to be localized preferentially in two organs, pancreas (4.82 ± 4% of total) and lung (4.43 ± 2.3% of total). 18.5 ± 4% of MSC derived in culture from these pancreata were found to be GFP positive. 

However once again, in absence of data on pancreatic tissues after bone marrow transplantation in humans, we can wonder how these results would be translatable to the human situation. In a study of a pancreas allograft removed 8 months after transplantation, it was found that part of pancreatic MSC expressed recipient HLA, suggesting an extrapancreatic origin of these cells [109]. 

Besides their putative differentiation capacity, MSC display very interesting additional characteristics. Sordi et al. reported that pancreatic MSC extracted from both endocrine and exocrine tissue and cotransplanted in mice with a minimal pancreatic islet mass facilitated the restoration of normoglycemia and neovascularization of the islet graft [109]. 

In conclusion, MSC can be derived from human exocrine tissue but show limited capacity of differentiation towards the endocrine pathway. Similarly to islet(-derived) MSC, the origin and role of these cells are unclear.

### 3.3. Extrapancreatic Sources of MSC

Bone marrow, adipose tissue, and umbilical cord blood are the main sources of BM-MSC reported so far. Hess et al. reported that transplantation of murine bone marrow cells in streptozotocin-treated mice was able to reduce hyperglycemia by initiating endogenous pancreatic regeneration. A majority of bone-marrow-derived cells were found to be localized near ductal and islet structures. Quantitative analysis of the pancreas revealed a very low frequency of donor insulin-positive cells. However the presence of donor cells was accompanied by a rapid proliferation of recipient pancreatic *β*-cells and by neogenesis of insulin-positive cells of recipient origin within a week after transplantation. The mechanism behind this regeneration process remains to be clarified. The authors suggested that bone-marrow-derived endothelial cells could be involved by secreting factors that enhance tissue repair [117]. This model was confirmed by others [118]. Along the same line, Lechner et al. observed no evidence for significant transdifferentiation of labeled (GFP transgenic mice) murine bone marrow into pancreatic *β*-cells *in vivo* [119]. By using a mouse model for impaired bone-marrow-derived cell mobilization (Nos3 −/− mice), Hasegawa et al. demonstrated that homing of donor bone-marrow-derived cells in recipient bone marrow and subsequent mobilization into the injured periphery were required for *β*-cell regeneration. Interestingly, simple bone marrow cell infusion without preirradiation had no effects, suggesting that injury signals are involved in triggering this process [120]. Finally, in similar transplantation experiments, Urban et al. revealed that murine-bone-marrow derived mesenchymal stem cells cooperate with bone marrow cells since neither bone marrow cells nor MSC transplantation was effective alone [121]. In contrast to all these studies and using a lineage tracing system (Ins/CRE-LoxP/EGFP), Ianus et al. claimed that transplanted murine bone marrow cells were able to differentiate themselves into functional *β*-cells [122]. Four to six weeks after transplantation from male mice into lethally irradiated recipient female mice, recipient mice contained Y chromosome and EGFP double-positive cells in their pancreatic islets. Of note, *β*-cell specificity was verified since neither bone marrow cells nor circulating peripheral blood nucleated cells of donor or recipient mice had any detectable EGFP. Cell fusion was also ruled out in this experiment. 

Regarding human cells, Prockop and colleagues evaluated the competence of bone marrow MSC in a similar type of experiment. Human bone marrow MSC were delivered via intracardiac infusions in diabetic NOD/SCID mice. Although rare *β*-cells were found to be of human origin (i.e., BM-MSC derived), blood glucose levels were found to be decreased after some weeks. The authors suggested that human BM-MSC were able to home to and promote repair of pancreatic islets and renal glomeruli in a diabetic mouse model [123]. In a parallel study, Sordi et al. showed that human BM-MSC express a restricted set of functionally active chemokine receptors (CXCR4, CX3CR1, CXCR6, CCR1, CCR7) capable of promoting migration to pancreatic islets [124]. Butler and collaborators studied 31 human pancreases obtained at autopsy from patients who had received a bone marrow-derived graft (26 cases), a peripheral blood-derived graft (4 cases), or a combination of both peripheral blood- and bone marrow-derived stem cells (1 case) [125]. More than 4000 islets were examined in this relatively large cohort, and no pancreatic *β*-cells were found to be derived from donor cells (including two cases of patients with type 2 diabetes). Therefore, it appears that in humans the bone marrow compartment does not contribute to pancreatic *β*-cell mass maintenance in healthy individuals. Nevertheless promising clinical results on glucose metabolism were recently reported. A clinical trial in 11 patients with type 1 diabetes was designed to test the safety and efficacy of intraportal coinfusion of insulin-producing adipose tissue-derived MSC and bone marrow cells. Differentiation of adipose tissue-derived MSC was initiated *in vitro* by a 3-day culture period in a defined medium described earlier [126]. A mean follow-up of about two years showed significant improvements of all clinical parameters related to diabetes (a decrease in insulin requirements, an increase in C-peptide levels, and absence of diabetic ketoacidosis) [127]. In a clinical trial involving 25 patients with type 2 diabetes, Ricordi and collaborators observed reduced insulin requirements and significant improvements of all metabolic variables (12-month follow-up) after an intrapancreatic infusion of autologous bone marrow cells [128]. However no data are available about the possible differentiation of MSC after implantation, and it is more likely that improved glucose metabolism is related to paracrine effects of MSC in this last study. 

*In vitro*, several groups reported the successful derivation of functional *β*-cells from rodent bone marrow cells in the presence or absence of serum [129] and with addition of growth factors like nicotinamide and *β*-mercaptoethanol [130] and conophylline and betacellulin-delta4 [131]. After transplantation these cells can (partly) revert hyperglycemia in diabetic mice. Also murine adipose tissue-derived MSC could be efficiently converted reaching up to 48% of cells that expressed c-peptide, following a 3-stage and 10-day differentiation protocol involving activin A, sodium butyrate, *β*-mercaptoethanol, taurine, GLP-1, nicotinamide, and nonessential amino acids [132].

The approach is slightly different in human cells, involving the combination of defined medium and virus-mediated ectopic expression of proendocrine transcription factors. Karnieli et al. reported that overexpression of Pdx1 in BM-MSC from 9 of 14 donors can trigger their differentiation to a *β*-cell-like phenotype displaying about 1% of the regular insulin content and able to control the insulin release in a glucose-dependent manner *in vitro* [133]. The cells lacked expression of Beta2/NeuroD1. However transplantation into streptozotocin-treated mice resulted in further differentiation, including induction of Beta2/NeuroD1 and reduction of hyperglycemia. Similar results were obtained in a parallel study [134]. Human MSC from other sources than bone marrow were also evaluated. MSC isolated from the Wharton's jelly of the umbilical cord were differentiated to islet-like cell clusters through stepwise culturing in neuron-conditioned medium [135]. The clusters were found to express islet-specific genes and to be glucose responsive *in vitro*. Functionality was further verified after transplantation into the liver of streptozotocin-induced diabetic rats via laparotomy. The presence of characteristic secretory granules was observed by electron microscopy 12 weeks after transplantation. 

In summary, the capacity of extrapancreatic mesenchymal stem cells to differentiate to *β*-cells *in vivo* appears to be very limited. Nevertheless, several groups reported a successful differentiation to functional *β*-cell-like cells *in vitro* especially from human MSC. MSC appear to display unique migratory and secretory properties (growth factors, cytokines) that make them attractive as “helper” cells for tissue repair (improve engraftment, viability, function) (for review see [92, 136]).

## 4. Mechanisms for Maintenance of the Cellular Identity

A crucial aspect common to all putative cell sources will be to uncover the mechanisms that preserve and control cell identity in order to enable successful manipulation of adult cell plasticity in clinical settings. Up to now, most efforts to influence the cell identity were focused on direct gene expression (overexpression/downregulation of key transcription factors) and growth-factor-mediated activation of specific signaling pathways. A new era has started aiming at better understanding the processes that regulate gene expression. Chromatin accessibility is a determining factor blocking or facilitating expression of specific genes. Cell identity is regulated by epigenetic factors that tightly regulate the activation or repression of genes including genomic DNA methylation, histone modifications, and noncoding RNA regulation (for review please see [137, 138]). Dhawan et al. recently demonstrated that pancreatic *β*-cell identity is maintained by DNA methylation-mediated repression of Arx [139]. The question whether all MSC(-like cells) are equal was recently addressed. Mutskov et al. investigated the patterns of histone modifications over the insulin gene in human islets and IPC (the MSC-like population derived *ex vivo* from human islets) compared to HeLa and BM-MSC [140]. Although neither IPC nor HeLa nor BM-MSC express insulin, IPC showed significant levels of active chromatin modifications, similarly to human islets although at a more moderate level. The probable multiorigin of the IPC might obscure the interpretation of these results. However these epigenetic marks absent in the unrelated cell types (HeLa and BM-MSC) might be part of a general mechanism whereby tissue-derived precursor/stem cells are committed to a distinct specification. Non-coding RNA (such as siRNA (short interference RNA), miRNA (microRNA), and lncRNA (long non-coding RNA)) are emerging as key players in regulation of development [141]. Joglekar et al. demonstrated that the miR-30 family of miRNAs contribute to the regulation of the dedifferentiation of human fetal *β*-cells through epithelial-to-mesenchymal transition by negatively regulating the translation of mesenchymal genes [142]. 

Epigenetic reprogramming of cell types with shared developmental history could be an effective strategy for pancreatic *β*-cell replacement therapies. The cells may display some intrinsic commitment to become islets even during adulthood and might thus require fewer triggers to differentiate/transdifferentiate towards a *β*-cell lineage. Along this line, in the field of reprogramming to iPS, the notion of epigenetic “memory” inherited from the parental cell is coming forward. Bar-Nur et al. observed a preferential lineage-specific differentiation in iPS derived from human *β*-cells [143]. These new insights in gene regulation should help to exploit the full potential of adult cell plasticity in the perspective of cell replacement therapies to treat diseases such as type 1 diabetes.

## 5. Conclusions and Discussion

In conclusion, it emerges that extreme caution should be taken when translating the findings obtained from rodent studies to the human situation. Regarding *in vivo* regeneration investigated under physiological conditions or under injury, it appears that *β*-cell replication is the predominant mechanism occurring in mice. In humans, this process seems to be restricted to the very early postnatal life, whereas during adulthood, neogenesis from (a subpopulation of) ductal and/or acinar cells seems to be responsible for the increase of *β*-cell mass required in physiological situations of higher insulin demands like obesity or pregnancy. Nevertheless, the unanswered question remains which cell type could be involved: progenitor cells present in the ductal area, or fully differentiated adult cells able to transdifferentiate, or a distinct subpopulation that is able to dedifferentiate to a progenitor-like intermediate stage followed by redifferentiation to an endocrine lineage. Remarkably, both the animal model and the degree of destruction appear to be key points, as the regeneration processes can be different: *β*-cell mass regeneration from *α* cells has been observed in models of near total ablation only. Regarding *in vitro* replicative capacity of *β*-cells, human *β*-cells cannot efficiently replicate, in contrast to rodent cells. However, contrary to murine *β*-cells again, human *β*-cells can dedifferentiate to a mesenchymal-like phenotype and proliferate. These discrepancies between findings from human versus rodent studies are not as unexpected as they seem, given the major differences observed in islet cell biology field such as timing of pancreatic developmental stages [45], islet architecture and composition [144, 145], and islet innervation [146]. In the stem cell field, murine and human stem cells are notoriously dissimilar. Finally, cultured human primary cells are more prone to replicative aging than murine cells as telomere shortening limits cell replication and leads to senescence.

However, up to now, there is no way to accurately evaluate the *β*-cell mass in humans, neither by imaging techniques nor by physiological measurements. Studies are limited to histological analysis performed on organs at autopsy or after pancreatectomy generating static pictures that could be misinterpreted, even if careful quantification and repeated observations in different contexts can still result in strong indications. Therefore, animal models become essential offering access to a broad range of technologies obviously not applicable to humans. Among others, the recently developed genetic approaches (such as lineage tracing systems, comprising of a tissue-specific promoter and a (tamoxifen-)inducible recombination system and a reporter gene) are valuable tools to follow a dynamic process and reinforce (or sometimes challenge) earlier theories suggested by more descriptive immunohistochemistry data. Nevertheless several limitations have to be taken into consideration: a possible leakiness of the recombination system, the relative specificity of a given promoter that can display some (transient) activity in nonspecific cells, and a limited penetrance (usually less than half of the cells are actually labeled) [147, 148]. Altogether these elements might contribute to the discrepancies between results obtained by different labs about the origin of *β*-cell regeneration for instance, and hopefully validation of some of the models by separate labs in different experimental contexts will clarify the situation in a near future. 

Since stimulation of human *β*-cells replication is still elusive, other cell sources have been envisaged. Studies from the last decade revealed an unexpected aspect of plasticity from mature differentiated cells by dedifferentiation or transdifferentiation. Of interest, it appears that regeneration does not always require recapitulation of the embryological development as needed for efficient differentiation of ES cells. For instance, the recent discovery of *α*-to-*β*-cell plasticity does not appear to correlate with any developmental process [7]. Murine and human ductal cells, rodent acinar cells (human ones cannot be maintained in culture), and human liver cells could be efficiently converted to functional *β*-cells able to revert hyperglycemia in a diabetic mouse model. Although the potential contribution of MSC to islet regeneration in a physiological situation remains unclear and their origin and function are still elusive, islet-derived mesenchymal stem cells seem to display specific genetic and epigenetic marks that could make them more prone to differentiation towards the endocrine compartment than extrapancreatic sources of MSC. This suggests that all mesenchymal stem cells (-like cells) are not equal. In addition MSC, in particular from humans, revealed additional properties (like homing to injury site and secretion of favorable growth factors) that may be of clinical use. Therefore, further investigations will be required to determine if islet-(derived-) MSC can be stimulated to contribute in any way to *β*-cell mass regeneration. Another approach successfully tested has been to force expression of proendocrine transcription factors *in vivo* in the liver and in the exocrine tissue. However no lineage tracing experiments have been performed in these studies, and the origin of the newly formed *β*-cells needs to be identified.

Finally, there is currently no unique optimal alternative cell source for *β*-cell (re)generation. Therefore a crucial aspect common to all putative cell sources will be to further uncover the mechanisms that preserve and control cell identity in order to enable successful manipulation of adult cell plasticity for clinical application.

## Figures and Tables

**Table 1:** Potential sources of de novo *β*-cells among differentiated adult cell types.

	Rodent		Human	
	*in vitro*	*in vivo*	*in vitro*	*in vivo*
*Endocrine*				
*β* -cells	+++	+++	−	−/+
*α*-cells	?	++	?	?

*Exocrine*				
Ductal cells	++	+	+	++
Acinar cells	+	−/+	?	?

*Extra-pancreatic*				
Liver cells	?	++	+	?

**Table 2:** Potential sources of de novo *β*-cells among differentiated adult MSC: BM: bone marrow, AT: adipose tissue; UCB: umbilical cord blood.

	Rodent		Human	
	*in vitro*	*in vivo*	*in vitro*	*in vivo*
*Pancreatic*				
Islet-(derived) MSC	?	?	+	?
Exocrine-(derived) MSC	?	?	−/+	?

*Extra-pancreatic*				
BM-MSC AT-MSC UCB-MSC	+	−/+	−/+	−
